# The Genetic Dissection of *Ace2* Expression Variation in the Heart of Murine Genetic Reference Population

**DOI:** 10.3389/fcvm.2020.582949

**Published:** 2020-11-20

**Authors:** Fuyi Xu, Jun Gao, Undral Munkhsaikhan, Ning Li, Qingqing Gu, Joseph F. Pierre, Athena Starlard-Davenport, Jeffrey A. Towbin, Yan Cui, Enkhsaikhan Purevjav, Lu Lu

**Affiliations:** ^1^Department of Genetics, Genomics and Informatics, University of Tennessee Health Science Center, Memphis, TN, United States; ^2^Department of Pediatrics, University of Tennessee Health Science Center, Memphis, TN, United States; ^3^Children's Foundation Research Institute, Le Bonheur Children's Hospital, Memphis, TN, United States; ^4^Department of Cardiology, Second Affiliated Hospital, Harbin Medical University, Harbin, China; ^5^Department of Cardiology, The Affiliated Hospital of Nantong University, Nantong, China; ^6^Pediatric Cardiology, St. Jude Children's Research Hospital, Memphis, TN, United States

**Keywords:** BXD mice, cardiovascular, ACE2, gene regulaiton, COVID-19

## Abstract

**Background:** A high inflammatory and cytokine burden that induces vascular inflammation, myocarditis, cardiac arrhythmias, and myocardial injury is associated with a lethal outcome in COVID-19. The SARS-CoV-2 virus utilizes the ACE2 receptor for cell entry in a similar way to SARS-CoV. This study investigates the regulation, gene network, and associated pathways of ACE2 that may be involved in inflammatory and cardiovascular complications of COVID-19.

**Methods:** Cardiovascular traits were determined in the one of the largest mouse genetic reference populations: BXD recombinant inbred strains using blood pressure, electrocardiography, and echocardiography measurements. Expression quantitative trait locus (eQTL) mapping, genetic correlation, and functional enrichment analysis were used to identify *Ace2* regulation, gene pathway, and co-expression networks.

**Results:** A wide range of variation was found in expression of *Ace2* among the BXD strains. Levels of *Ace2* expression are negatively correlated with cardiovascular traits, including systolic and diastolic blood pressure and P wave duration and amplitude. *Ace2* co-expressed genes are significantly involved in cardiac- and inflammatory-related pathways. The eQTL mapping revealed that *Cyld* is a candidate upstream regulator for *Ace2*. Moreover, the protein–protein interaction (PPI) network analysis inferred several potential key regulators (*Cul3, Atf2, Vcp, Jun, Ppp1cc, Npm1, Mapk8, Set, Dlg1, Mapk14*, and *Hspa1b*) for *Ace2* co-expressed genes in the heart.

**Conclusions:** *Ace2* is associated with blood pressure, atrial morphology, and sinoatrial conduction in BXD mice. *Ace2* co-varies with *Atf2, Cyld, Jun, Mapk8*, and *Mapk14* and is enriched in the RAS, TGFβ, TNFα, and p38α signaling pathways, involved in inflammation and cardiac damage. We suggest that all these novel Ace2-associated genes and pathways may be targeted for preventive, diagnostic, and therapeutic purposes in cardiovascular damage in patients with systemic inflammation, including COVID-19 patients.

## Introduction

Angiotensin (Ang) converting enzyme 2 (ACE2) is a protein found as both a membrane-associated and secreted enzyme in cardiac, vascular, pulmonary, neuronal, and reproductive organs (1). In the renin-angiotensin-aldosterone system (RAS or RAAS), ACE2 catalyzes the conversion of AngII to Ang1–7, which acts as a vasodilator (2) and plays a critical role in the control of cardiovascular and renal functions by maintaining the physiological homeostasis of blood pressure (BP) and electrolyte balance (3). ACE2, in turn, contributes to the cardioprotective effects of Ang1–7 after myocardial infarction (4), ischemic cardiomyopathy (5), and myocardial ischemia/reperfusion (6). Moreover, ACE2 counterbalances with ACE and functions as a negative regulator of the RAS (7), playing a critical protective role against heart failure with reduced and preserved ejection fraction, myocardial infarction, and hypertension as well as against lung disease and diabetes (8). Knockout of ACE2 in mice resulted in severe cardiac contractile deficiency due to increased AngII levels and upregulation of hypoxia-induced genes in the heart, suggesting that ACE2 is an essential regulator of cardiac function (9). However, the full spectrum of underlying genetic pathways and networks of ACE2 in the heart remains to be understood.

Coronavirus disease (COVID-19), caused by a novel severe acute respiratory syndrome coronavirus 2 (SARS-CoV-2), is a recent global pandemic infectious disease with almost unprecedented speed of spread from an initial outbreak in November or December 2019 (10). SARS-CoV-2 causes primary pneumonia while also causing acute myocardial injury and chronic damage to the cardiovascular system (11). In addition, COVID-19 is associated with a high inflammatory burden that can induce vascular inflammation, myocarditis, and cardiac arrhythmias (12). In particular, inflammation and cytokine surge may be a potential mechanism for myocardial injury (13). Similar to SARS-CoV, SARS-CoV-2 uses ACE2 as the receptor to enter the host cell facilitating the viral endocytosis (14, 15). Therefore, the ACE2 expression pattern in cardiac tissue may play a critical role in the cardiovascular damage from the SARS-CoV-2 infection, and elucidation of its regulatory mechanisms would be beneficial for preventive, diagnostic, and therapeutic purposes of cardiovascular damage in COVID-19.

BXD recombinant inbred (RI) strains, one of the largest and best-characterized mouse genetic reference populations (GRPs) currently, have been constructed for systems genetics study in unraveling the genetic architecture of polygenic traits. Currently, ~152 BXD progeny strains derived from crosses between C57BL/6J (B6) and DBA/2J (D2) parents have been generated and maintained in our lab (16). Both parental strains and BXD progeny strains have been sequenced, which showed that they segregate more than 6 million variants, similar to humans with a mean minor allele frequency close to 0.5 (17). Each BXD strain with the same genetic background can be replicated in large numbers per genome as a murine GRP for systems genetics analysis, enabling uncovering candidate genes and mechanisms related to complex traits. It facilitates more precise phenotypic estimates for mapping complex traits with low-to-moderate heritability as well (16). We have successfully used our BXD cohort for identification of candidate genes in hypertension, cardiac and renal damage, and cardiac fibrosis (18–21).

In this study, we aimed to assess the expression variation of *Ace2* in the heart tissue of BXD mice and the association with cardiovascular traits. Through the systems genetics approach, we sought to identify the *Ace2*-correlated genes, potential pathways, and candidate upstream or downstream regulators that may contribute to cardiovascular damage upon systemic inflammatory background.

## Materials and Methods

### Heart Expression Data Set

The gene expression data, EPFL/LISP BXD CD Heart Affy Mouse Gene 2.0 ST (Jan14) RMA, used in this study was generated through collaborative efforts and can be accessed at our GeneNetwork (GN) website (http://genenetwork.org/) with the GN485 accession number (22).

#### Mice

Male mice of 40 BXDs and their two founder strains B6 and D2, representing murine GRP, were used in this study. All mice were fed a chow diet (6% kcal/fat, 20% protein, 74% carbohydrate, Harlan, 2918) throughout life after weaning until sacrifice at around 29 weeks of age. For cardiac tissue collection, animals were sacrificed under isoflurane anesthesia after an overnight fast. All animal procedures were approved by the Swiss cantonal veterinary authorities of Vaud under licenses 2257.0 and 2257.1.

#### RNA Isolation and Microarray

Hearts were later shattered in liquid nitrogen, usually broken into 2–4 pieces, and around half of the sample (at random) was taken for preparation. Total RNA was isolated using the Trizol reagent (Invitrogen, Carlsbad, CA). RNA samples from ~5 mice of the same strain were pooled equally (by microgram of RNA) into a single RNA sample. The pooled RNA samples were then purified using RNEasy (Qiagen, Hilden, Germany). Samples that passed quality control (RIN > 8.0) were run on Affymetrix Mouse Gene 2.0 ST in a single batch at the University of Tennessee Health Science Center (UTHSC).

#### Data Preprocessing

Raw microarray data were first normalized using the robust multichip array (RMA) method (23), and then the data were logged and Z normalized (24). Instead of leaving the mean at 0 and the standard deviation of 1 unit, we shift up to a mean of 8 units and increase the spread by having a standard deviation of 2 units (what we call 2Z + 8 normalized data). This removes negative values from the tables.

### Cardiac Traits Measurement

The cardiovascular system–related phenotype was collected in 3- to 4-month-old mice. This included 94 mice (50 strains) for collection of 18 electrocardiogram (ECG) traits, 425 mice (45 strains) for 16 echocardiogram traits, and 424 mice (24 strains) for 5 BP traits. The CODA non-invasive tail-cuff BP system with a volume pressure recording (VPR) sensor (Kent Scientific Corporation) measured systolic and diastolic BP. Before measuring BP, mice were acclimated to the restraint holder placed on a heating pad for 3 days. For BP recording, the VPR cuff was placed at the base of the tail, and sessions consisted of 15 inflation and deflation cycles with the first five being acclimation cycles. At least 5 cycles per mouse were used in the analysis. Transthoracic echocardiography was used to evaluate heart function by short and long cross-sectional, two-dimensional, color Doppler using a Vevo2100 Micro-Imaging System (VisualSonics Inc., Toronto, Canada) with a 30-MmHz transducer. Single-lead ECG tracings were recorded for 5 min at a sampling rate of 200 Hz using BIOPAC (Goleta, CA USA) with AcqKnowledge 3.9.2 software. Normal and arrhythmic heart rhythms were distinguished relying on the minimum and maximum RR intervals using LabChart 7 software.

### eQTL Mapping

Two methods were used for eQTL mapping for *Ace2* expression on GN, the fast linear mapping (25) method and the genome-wide efficient mixed model association (GEMMA) method (26). Fast linear mapping used the likelihood ratio statistics (LRS) to measure the linkages between the investigated phenotype (clinical or intermediate traits) and genotype markers. The genome-wide significant and suggestive QTLs were determined with 2,000~10,000 permutation tests (suggestive LRS = 11.3 and significant LRS = 18.4). For GEMMA, a linear mixed model method was used to effectively correct the kinship among samples. In addition, it incorporates the leave one chromosome out (LOCO) method to ensure that the correction for kinship does not remove useful genetic variance near each marker. The suggestive and significant threshold for a genome-wide scan is –log (p) of 2.5 and 4.0. The threshold was based on one unit of –log(p) being roughly equivalent to 1 unit of logarithm of the odds (LOD) value, where LOD = LRS/4.61. Both methods used a total of 7,321 informative SNP genotype markers for the analysis. The BXD genotype file can be accessed and reviewed on GN.

### Correlation Analysis

Genetic correlation analysis was performed with the Pearson correlation coefficient to identify gene–gene and gene–phenotype relationships. The literature correlation quantifies genes that are described by similar terminology in published papers extracted from MEDLINE/PubMed abstracts. Similarity values, known as literature correlations, are computed for a matrix of about 20,000 genes using latent semantic indexing (27, 28). These values are always positive and range from 0 to 1. Both analyses were done on GN.

To evaluate the false discovery rate (FDR) for gene–gene, we computed the adjusted *p-*value with Westfall and Young's multiple testing procedure (29). Briefly, we randomly permuted the *Ace2* expression data 1,000 times. For each permutation, we computed the *p-*value of Pearson's correlation between the randomized *Ace2* and the other cardiac transcripts (41,355 probe sets). The adjusted *P-*value was determined by ranking the correlation coefficient. For gene–phenotype relationships, the same analysis was done between the randomized *Ace2* and phenotypes as well as between the randomized *Cyld* and phenotypes in each cardiac trait categories (BP, ECG, and echocardiogram).

### Gene Enrichment Analysis

Genes with significant correlations with *Ace2* (FDR <0.05 and *r* > 0.3) were used for gene set over representation analysis for Kyoto Encyclopedia of Genes and Genomes (KEGG) pathway and Mammalian Phenotype Ontology (MPO) on the Webgestalt website (http://bioinfo.vanderbilt.edu/webgestalt/) (30). Mouse protein-coding genes were used as a reference gene set with a minimum of 5 genes for each category.

### Genetic Variations

Genetic variations between the founder strains B6 and D2 were obtained from whole genome sequencing analysis in our previous publication (31). Here, we focused on the variants located in the coding sequence region, such as stop gain, stop loss, frameshift, and missense variants.

### Exploration of Gene Function

In order to identify genes with functions related to inflammation and the cardiovascular system. We retrieved lists of genetically engineered alleles, transgenes, or QTL variants from The Phenotypes/Alleles project (http://www.informatics.jax.org/allele) in Mouse Genome Informatics (MGI) (32) with the key words “inflammation” or “cardiovascular.” This portal enables access to spontaneous, induced, and genetically engineered mutations and their strain-specific phenotypes.

### Candidate Gene Selection

For the *Ace2* modulating eQTL, all positional candidates in the confidence interval were selected for further analysis based on the following criteria: (1) mRNA correlation with *Ace2*, including genetic correlation (FDR <0.05) and literature correlation (*r* > 0.3); (2) presence of *cis* regulation, defined as LRS > 12 for the peak SNP within the 5-Mb interval of its gene's physical position; (3) presence of coding sequence variation between B6 and D2; (4) mRNA correlation with the cardiovascular traits; and (5) gene function related to inflammation or cardiovascular system.

### PPI Network

In order to further discover the key regulators in *Ace2* co-expressed genes, we created and evaluated the PPI network with the NetworkAnalyst (www.networkanalyst.ca) (33), in which the International Molecular Exchange (IMEx) Interactome database was used (34). The IMEx consortium is a publicly available database of curated and non-redundant set of protein interactions.

## Results

### Levels of *Ace2* mRNA in the Heart Are Significantly Correlated With Cardiovascular Traits

**Figure 1:**
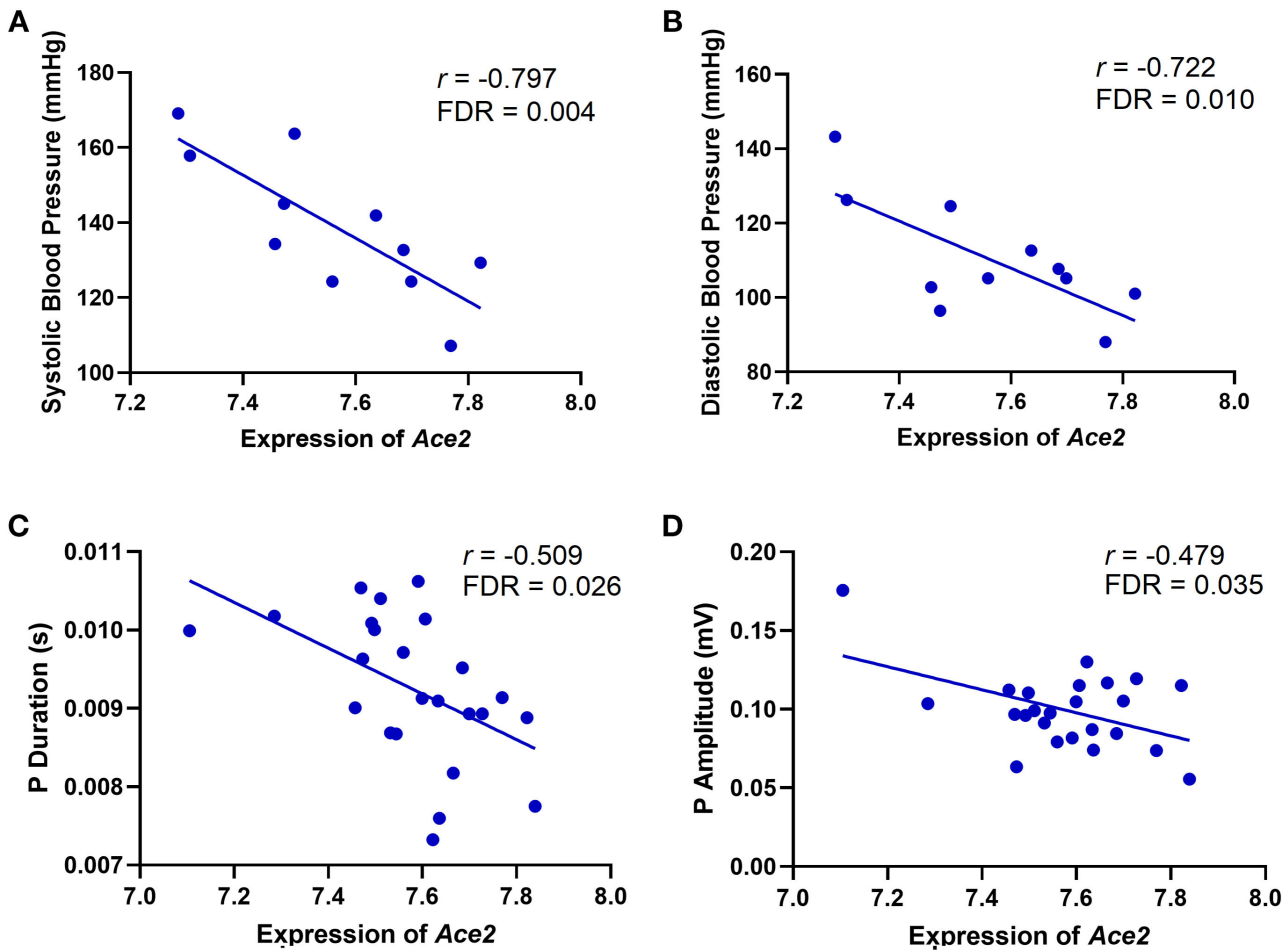
Scatterplots of the correlations of *Ace2* expression with systolic BP **(A)**, diastolic BP **(B)**, P duration **(C)**, and P amplitude **(D)**. The Pearson correlation coefficient was used to determine the relationship. Pearson correlation *r* and FDR are indicated. Gene expression levels are log2 transformed.

*Ace2* has been reported to be associated with several cardiovascular system–related phenotypes under various mouse genome backgrounds (9, 35–37). To define an association between *Ace2* and cardiovascular traits in the BXD mice, we performed Pearson correlation analysis of *Ace2* mRNA level against cardiovascular traits collected by BP measurements, ECG tracings, and transthoracic echocardiography. Results show that *Ace2* expression is significantly and negatively correlated with both systolic (*r* = −0.797, FDR = 0.004, Figure 1A) and diastolic (*r* = 0.722, FDR = 0.010, Figure 1B) BP, indicating that higher *Ace2* levels were observed in BXD strains with lower systolic and diastolic pressure. Further, *Ace2* has a negative association with the P duration (*r* = −0.509, *p* = 0.011, FDR = 0.026, Figure 1C) and P amplitude (*r* = −0.479, FDR = 0.035, Figure 1D), indicating that BXD strains showing narrower and lower P waves on ECG tracings have higher *Ace2* expression. P-wave amplitude and duration changes together have important diagnostic values in hemodynamic and morphological changes of the left atrium (LA) and sinoatrial conduction in patients with hypertension, left ventricular (LV) hypertrophy, and diastolic dysfunction (38). Thus, we calculated a Pearson correlation and found no association between BP and P-wave amplitude and duration in BXDs (*p* = 0.61 and 0.65 for systolic/diastolic BP vs. P-wave amplitude; *p* = 0.30 and 0.31 for systolic/diastolic BP vs. P-wave duration). Further, we found no association between *Ace2* expression and echocardiographic parameters of LV function, including EF%, FS%, and LV volumes or wall thickness in BXD strains. Collectively, phenotype–genotype correlation predicted a strong association of *Ace2* with the genes regulating BP as well as atrial morphology and sinoatrial conduction while no association was predicted between *Ace2* and LV dysfunction.

### eQTL Mapping Identifies an *Ace2* Regulating Locus on Chromosome 8

**Figure 2:**
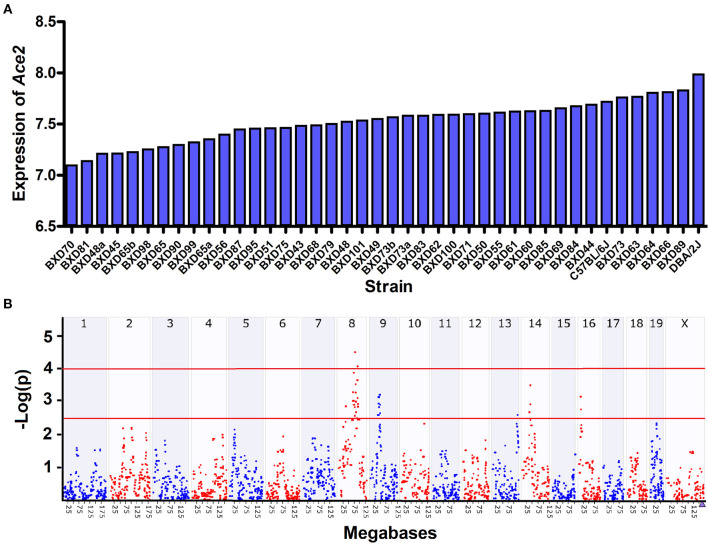
Expression and eQTL mapping of *Ace2* in BXD family. **(A)** Bar plots of the *Ace2* expression levels of heart across the BXD mice. The x-axis shows the BXD strains and the two parental strains. The y-axis shows the normalized log2 expression levels of *Ace2*. **(B)** Manhattan plot of genome-wide *Ace2*-regulated genomic loci. The x-axis denotes a position on the mouse genome, in megabases (Mb), while the y-axis gives the –log (p), a measurement of the linkage between *Ace2* expression, and genomic region. The purple triangle indicates the genomic position of *Ace2*. The red line indicates suggestive (–log (p) of 2.5) and significant (–log (p) of 4.0) genome-wide thresholds. eQTL mapping was conducted with GEMMA on GN.

We observed a 1.8-fold change of the *Ace2* expression across the 40 BXD strains and their parents (Figure 2A). The average expression of *Ace2* across the strains is 7.53 ± 0.20 SD. BXD70 and D2 mice have the lowest and highest expression of 7.1 and 8.0, respectively.

We then looked up the genetic variations in *Ace2* in the two founder strains across our whole genome sequencing data. Although we did not find any protein-coding variants, three variants (*rs52428988, rs387358736*, and r*s230145272*) were located at upstream of *Ace2*. Next, we performed eQTL mapping for *Ace2* expression. One suggestive eQTL was mapped to Chromosome (Chr) 8 at 88.4 Mb using interval mapping methods (Peak SNP LRS = 15, suggestive LRS = 11.3, and significant LRS = 18.4), and achieved significance with GEMMA after correcting the kinship among samples (Figure 2B). This locus is apart from the *Ace2* genomic location (Chr X at 164.14 Mb), suggesting this is a *trans*-acting eQTL.

### *Cyld* Is a Candidate Upstream Regulator for *Ace2*

The 1.5-LOD interval for Chr 8 QTL encompasses 33 Mb from 63 to 96 Mb. Among the genes in the QTL region, we identified 17 genes (Table 1) that had significant genetic correlation (FDR <0.05) and literature correlation (*r* > 0.3) with *Ace2*. In order to further prioritize the candidates, we first performed eQTL mapping for those 17 genes. This resulted in the identification of 6 genes (*Jund, Il15, Nfix, Prdx2, Cyld*, and *Coq9*) that were *cis*-regulated. Moreover, by comparing the DNA sequence differences between the B6 and D2 mice, 5 genes (*Lpl, Clgn, Il27ra, Cc2d1a*, and *Ces1c*) were identified to harbor missense variants. Ten of the genes (*Klf2, Ciapin1, Jund, Il15, Coq9, Lpl, Cc2d1a, Dnase2a, Cyld*, and *Il27ra*) have been implicated in the cardiovascular system or inflammation response in various natural, induced, or genetically engineered mice according to the Phenotypes/Alleles project (http://www.informatics.jax.org/allele) in the MGI (32).

**Table 1:** Lists of the candidate genes in Chr 8 QTL interval.

**Gene ID**	**Symbol**	**Location (Chr: Mb)**	**Mean Expression**	***Ace2***			**Cis-eQTL**	**Coding variants**	**Cardiovascular**	**Inflammation**
				**Pearson *r***	**FDR**	**Literature *r***				
16956	*Lpl*	Chr8: 68.894625	10.93	−0.4	0.008	0.37	×	√	√	×
67184	*Ndufa13*	Chr8: 69.894180	13.57	−0.39	0.010	0.38	×	×	×	×
16478	*Jund*	Chr8: 70.697739	10.52	0.36	0.018	0.47	√	×	√	√
17274	*Rab8a*	Chr8: 72.180496	7.19	−0.41	0.006	0.35	×	×	×	×
16598	*Klf2*	Chr8: 72.319033	10	0.33	0.030	0.46	×	×	√	×
16168	*Il15*	Chr8: 82.331624	9.91	−0.33	0.030	0.31	√	×	√	√
12745	*Clgn*	Chr8: 83.389867	6.8	−0.32	0.036	0.31	×	√	×	×
81489	*Dnajb1*	Chr8: 83.608175	8.89	0.42	0.005	0.33	×	×	×	×
50931	*Il27ra*	Chr8: 84.030286	8.69	−0.47	0.001	0.37	×	√	×	√
212139	*Cc2d1a*	Chr8: 84.132828	8.79	0.39	0.010	0.33	×	√	√	×
18032	*Nfix*	Chr8: 84.699876	11.48	−0.37	0.015	0.33	√	×	×	×
13423	*Dnase2a*	Chr8: 84.908560	9.01	0.4	0.008	0.43	×	×	×	√
21672	*Prdx2*	Chr8: 84.969587	11.48	0.41	0.006	0.5	√	×	×	×
74256	*Cyld*	Chr8: 88.697028	9.44	0.46	0.002	0.38	√	×	×	√
13884	*Ces1c*	Chr8: 93.099015	7.32	−0.31	0.043	0.36	×	√	×	×
109006	*Ciapin1*	Chr8: 94.819818	11.59	0.35	0.021	0.33	×	×	√	×
67914	*Coq9*	Chr8: 94.838321	12.49	0.41	0.006	0.31	√	×	√	×

**Figure 3:**
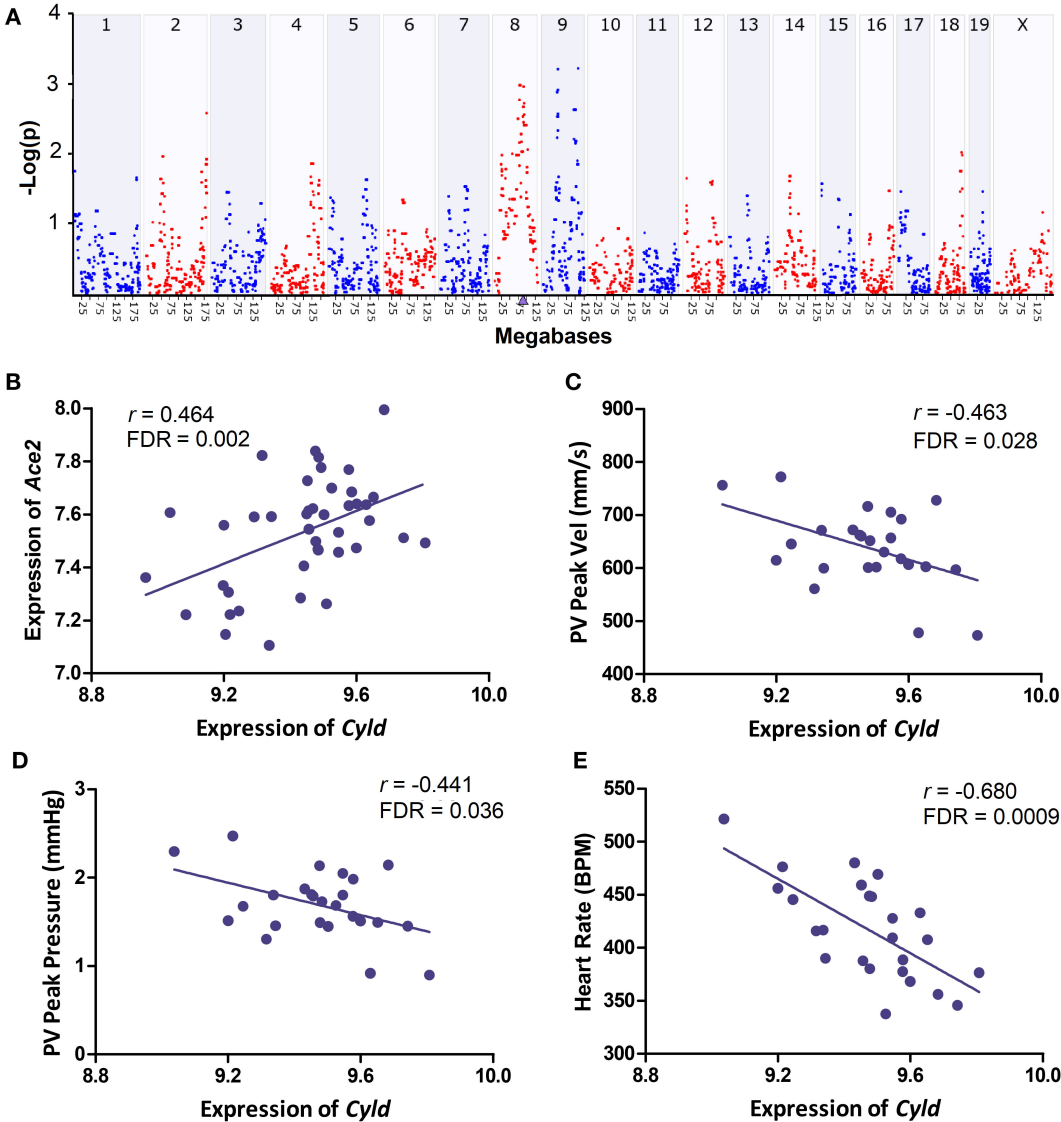
Genetic mapping and correlation analysis of *Cyld*. **(A)** Manhattan plot of genome-wide *Cyld* regulated genomic loci. The x-axis denotes a position on the mouse genome, in megabases (Mb), while the y-axis gives the –log(p), a measurement of the linkage between *Cyld* expression and genomic region. The purple triangle indicates the genomic position of *Cyld*. Genome-wide eQTL mapping was conducted with GEMMA on GN. **(B–E)** Scatterplots of the correlations of *Cyld* with *Ace2*, PV peak velocity (Vel), PV peak pressure, and heart rate. Pearson correlation coefficient was used to determine the relationship. Pearson correlation *r* and FDR are indicated. Gene expression levels are log2 transformed.

Based on the above criteria of candidate gene identification, we selected *Cyld* as the best candidate among the genes in this QTL interval. This gene encodes CYLD lysine 63 deubiquitinase and is located at the QTL peak position (Chr 8 at 88 Mb). Although *Cyld* harbors no coding sequence variants, more than 30 3′-UTR variants were unveiled between the two founder B6 and D2 strains, and eQTL mapping demonstrated that this gene is *cis*-regulated (Figure 3A). Besides, *Cyld* was positively correlated with *Ace2* expression (r = 0.46, FDR = 0.002, Figure 3B). Importantly, we found the levels of *Cyld* mRNA negatively correlated with echocardiography parameters, such pulmonary valve (PV) peak velocity (*r* = −0.463, FDR = 0.028, Figure 3C) and PV peak pressure (*r* = −0.441, FDR = 0.036, Figure 3D). These echocardiographic correlations suggest that the BXD strains with faster blood flow through PV and higher pressure on PV have significantly lower *Cyld* expression, suggesting that decreased *Cyld* expression is associated with elevated pulmonary artery pressure and pulmonary hypertension (PH). In addition, the levels of *Cyld* mRNA negatively correlated with heart rate (*r* = −0.680, FDR = 0.0009, Figure 3E) in BXD mice, showing that the BXD strains with lower *Cyld* expression have an increased heart rate.

### *Ace2* Co-expressed Genes Are Involved in Cardiac and Inflammatory Functions

**Figure 4:**
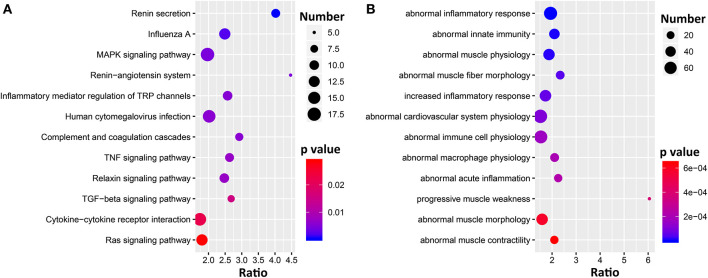
Bubble charts of the KEGG pathways **(A)** and MPO **(B)** enriched for *Ace2* co-variates. Gene over-representation analysis for KEGG pathway and MPO of the *Ace2* correlated genes (FDR <0.05 and *r* > 0.3) were performed with WebGestalt (http://www.webgestalt.org/). The x-axis represents an enriched ratio, and the y-axis represents enriched pathways/terms. The size of the dots represents the number of genes, and the color indicates the *p*-values. An enriched ratio is defined as the number of observed divided by the number of expected genes from the annotation category in the gene list.

To gain insight into the pathways and biological functions of *Ace2* involvement in the heart, we performed genetic correlation analysis for *Ace2* against the whole heart transcriptome. We identified a total of 2,800 transcripts that are significantly (FDR <0.05) correlated with the expression of *Ace2* in the heart, among which 440 genes shown literature correlation *r* > 0.3 with *Ace2*. Those genes were further submitted to WebGestalt (http://www.webgestalt.org/) for the KEGG pathway and MPO enrichment analysis. Results clearly demonstrated that the genes were overrepresented in two biological systems: the cardiovascular system as well as inflammation and immune response Figure 4. The KEGG pathways included renin secretion, mitogen-activated protein kinase (MAPK), RAS, tumor necrosis factor (TNF), and transforming growth factor beta (TGFβ) signaling pathways (Figure 4A). The MPO revealed abnormal inflammatory response, abnormal innate immunity, abnormal muscle physiology, and abnormal cardiovascular system physiology (Figure 4B).

**Figure 5:**
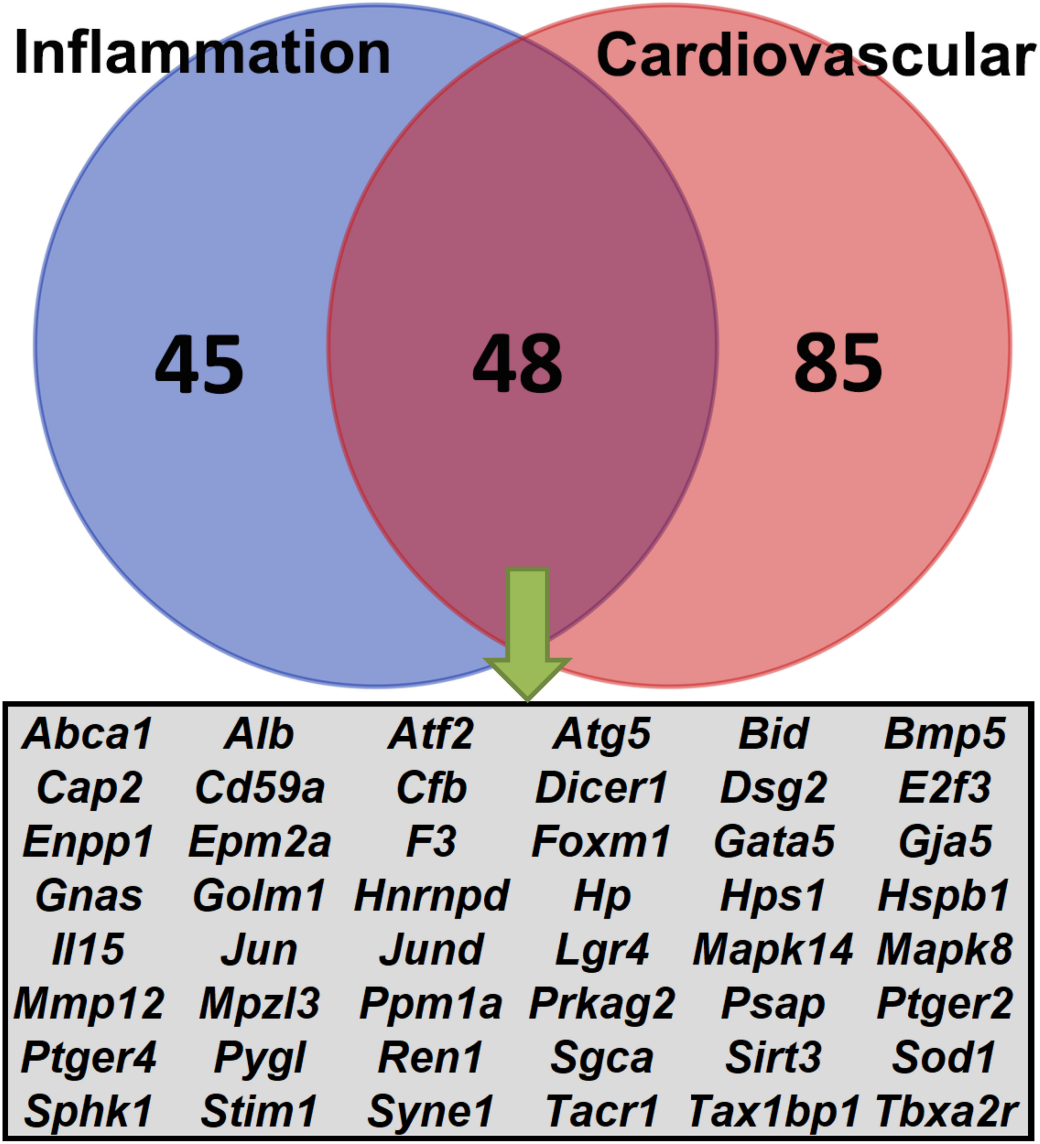
Venn diagrams of the *Ace2* correlated genes. A total of 440 genes were correlated (FDR <0.05 and *r* > 0.3) with *Ace2*. Lists of genetically engineered alleles, transgenes, or QTL variants were retrieved from the Phenotypes/Alleles project (http://www.informatics.jax.org/allele) in the MGI (32) with key words “inflammation” or “cardiovascular.” The Venn diagram shows the number of overlapping genes between the two categories.

Next, we focused on exploring the *Ace2* co-expressed genes in the two enriched biological systems, inflammation and cardiovascular, using the Phenotypes/Alleles project (http://www.informatics.jax.org/allele). This resulted in 90 and 133 genes with spontaneous, induced, or genetically engineered mutations that are associated with inflammation- or cardiovascular-related traits (Figure 5). It is worth noting that 48 genes (Figure 5) met the above two criteria, including genes *Dsg, Prkag2, Sgca, Syne1*, and *Ren1*.

### PPI Network Analysis Infers Key Regulators for *Ace2* Co-expressed Genes in the Heart

**Figure 6:**
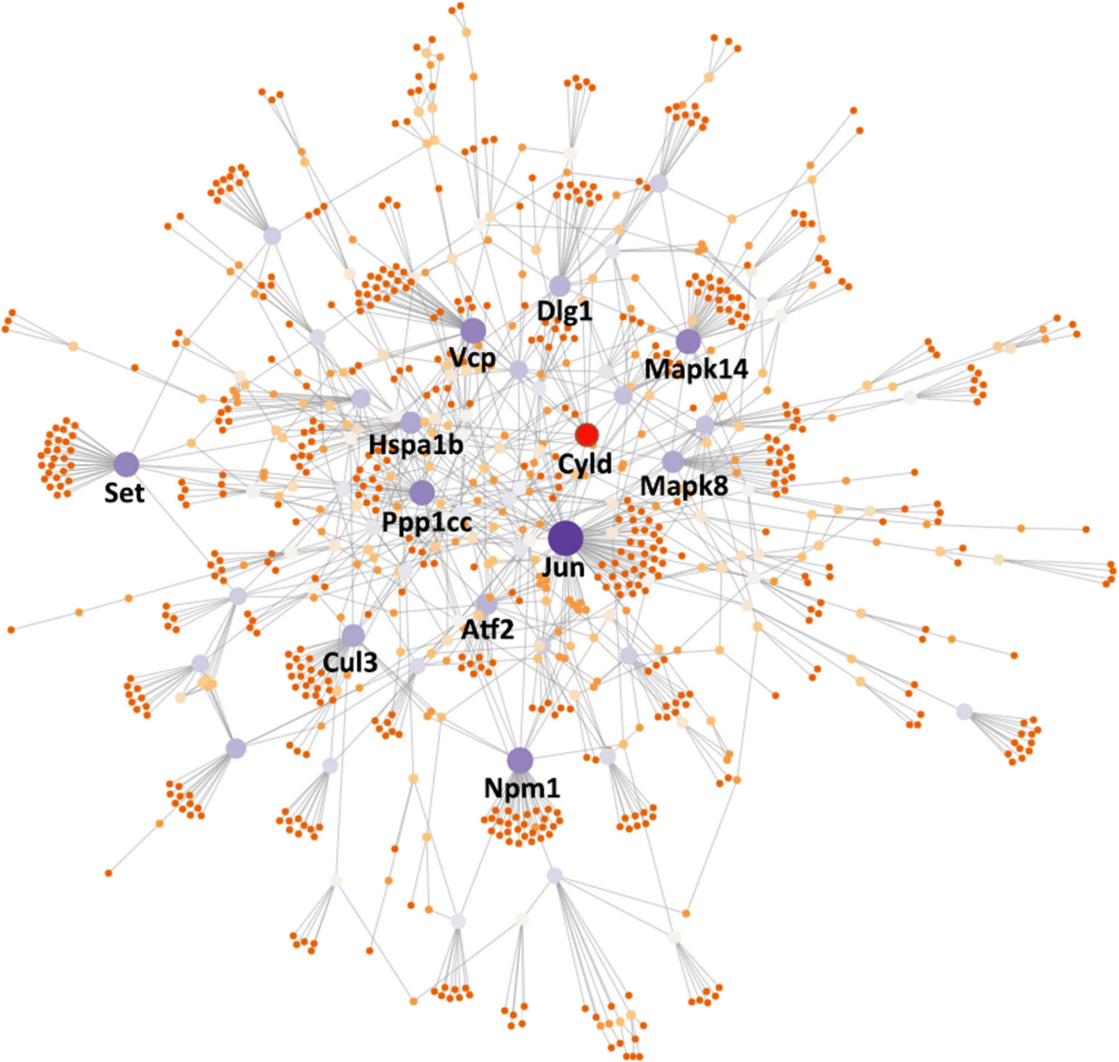
PPI network analysis inferred key regulators of *Ace2*. The PPI network was constructed using NetworkAnalyst (https://www.networkanalyst.ca/) with the input of *Ace2* co-expressed genes. The nodes in the network represent genes. Key node genes were indicated with gene symbols. Generic PPI was constructed using the IMEx Interactome database.

Finally, in order to investigate the key regulators among the *Ace2* co-expressed genes, we submitted the 440 transcripts that are significantly correlated with *Ace2* (FDR <0.05 and *r* > 0.3) to the NetworkAnalyst (www.networkanalyst.ca) (33) and evaluated the PPI network with the curated and non-redundant set of protein interactions in the IMEx consortium database (34). The PPI network suggests that 12 genes (*Cul3, Atf2, Vcp, Jun, Ppp1cc, Cyld, Npm1, Mapk8, Set, Dlg1, Mapk14*, and *Hspa1b*) are at the central node of the network and have the most connections to other genes (Figure 6). Notably, most of these genes are involved in the regulation of cardiovascular and inflammation-related traits (Table 2).

**Table 2:** List of 13 prioritized genes associated with the inflammation and cardiovascular traits.

**Gene ID**	**Symbol**	**Location (Chr: Mb)**	**Mean Expression**	***Ace2***		**Cardiovascular**	**Inflammation**
				**Pearson *r***	**FDR**		
26554	Cul3	Chr1: 80.264923	13.13	0.43	0.004	√	×
11909	Atf2	Chr2: 73.816509	10.38	0.31	0.043	√	√
269523	Vcp	Chr4: 42.979964	8.74	0.33	0.030	√	×
16476	Jun	Chr4: 95.049034	12.16	0.52	0.0004	√	√
19047	Ppp1cc	Chr7: 119.739914	12.68	0.31	0.043	×	×
74256	Cyld	Chr8: 88.697028	9.44	0.46	0.002	×	√
18148	Npm1	Chr11: 33.152287	8.29	0.32	0.036	√	×
26419	Mapk8	Chr14: 33.377898	10.56	0.39	0.010	√	√
56086	Set	Chr16: 15.451895	10.23	0.46	0.002	×	×
13383	Dlg1	Chr16: 31.665643	12.96	0.55	0.0001	×	×
26416	Mapk14	Chr17: 28.691342	10.85	0.4	0.008	√	√
15511	Hspa1b	Chr17: 34.956429	8.75	0.34	0.025	×	×

## Discussion

Accumulated evidence indicates that ACE2, a gatekeeper of RAS that coronaviruses use as a host functional receptor, is a potential therapeutic target due to its degrading effects on AngII in hypertension, LV hypertrophy, fibrosis, and diastolic dysfunction, including cardiovascular complication in COVID-19 (9, 39). The current pandemic COVID-19 infection has been associated with multiple cardiovascular complications, and at least 8.0% of COVID-19 patients suffer acute cardiac injury (40). Moreover, the high inflammatory burden of SARS-CoV-2 has been shown to induce vascular inflammation, myocarditis, and cardiac arrhythmias (13). Since a complicated picture has emerged on the association of ACE2 effects on cardiovascular physiology in the COVID-19 pandemic, we hypothesized that dissection of the cardiac genetic regulatory network of *ACE2* using a systems genetics approach would uncover the underlying genetic determinants in *ACE2*-driven cardiovascular function, the results of which would benefit in the diagnosis and care of patients with cardiovascular disease, including cardiovascular complications in COVID-19. In the present study, we collected cardiovascular traits in murine GRP of BXD strains and correlated those traits with their cardiac gene expression. Then, we identified co-expressed genes of *Ace2*, upstream and downstream regulators, networks, and pathways to uncover the underlying genetic determinants in *ACE2*-driven cardiovascular function and inflammation as well as immune response.

The important cardiovascular traits, systolic and diastolic BPs, are found to be significantly negatively correlated with *Ace2* expression in BXDs, demonstrating that the lowest *Ace2* levels are associated with hypertension. Supporting our results, Ace2 deficiency resulted in BP increase (9) while *Ace2* ablation also exacerbates AngII-mediated inflammation and myocardial injury resulting in cardiac dysfunction in Ace2 knockout mice (41). In the Yamamoto et al. (36) study, Ace2^−/y^ mice received transverse aortic constriction developed cardiac hypertrophy and dilatation, and this event was associated with significantly increased concentration in cardiac AngII and MAPK activity. In our study, *Ace2* expression is significantly and negatively correlated with the P-wave duration and amplitude in ECG tracings of BXD mice. These changes in P-wave were not associated with BP while *Ace2* expression had no association with echocardiography parameters of LV function. These results strongly suggest an underlying association of *Ace2* with the genes regulating BP as well as atrial function (diastolic dysfunction and atrial dilation) and sinoatrial conduction abnormalities.

To uncover the genetic correlates and pathways of Ace2, gene set enrichment analysis was performed, which found several *Ace2* correlated genes involved in two main KEGG pathway categories, such as RAS system-related (renin secretion, RAS, and RAS signaling pathway) and inflammation-related (inflammatory mediator regulation of TRP channels, MAPK, TNF, TGFβ, and cytokine–cytokine receptor interaction) signaling pathways (Figure 4A). The *Ren1* encoding renin, an RAS initiative enzyme (42), was found to be significantly associated with *ACE2*, emerging in two signals: renin secretion and the RAS KEGG pathway. In mice, some strains (e.g., C57BL/6) have only *Ren1*, whereas others (e.g., DBA/2, J129) have two, *Ren1* and *Ren2*, genes (43). An increase in *Ren1*, in particular, causes a striking overexpression of renin in kidney tubules *in vivo*, partially explaining the association between *Ace2* and BP in BXDs (44). For the inflammation pathway, TNFα is a master cytokine that mediates inflammation and innate immune responses (45). MAPKs not only regulate TNFα expression by several mechanisms (45), but also involved in different facets of cardiac hypertrophy, cardioprotection *vs*. myocardial cell death or cardiac remodeling (46). Through the PPI network analysis, we elucidated the key regulators of the Ace2 co-expressed genes on a protein level involved in heart function, such as *Atf2, Cul3, Cyld, Dlg1, Hspa1b, Jun, Mapk8, Mapk14, Npm1, Ppp1cc, Set*, and *Vcp*. Among those genes, *Mapk8* (also known as *Jnk1*) and *Mapk14* (also known as *p38*α) belong to the MAPK family. *Jnk1* encodes four different isoforms of c-Jun N-terminal kinases (JNK) (47) involved in inflammation (48), and it also has been implicated in cardiac fibrosis and cardiomyocyte apoptosis (49). In addition, JNK alters the activity of many proteins that reside in mitochondria or act on the nucleus through phosphorylation, including activating transcription factor 2 (ATF2), one of the node genes found in our PPI network. *Mapk14* is a critical regulator of vascular smooth muscle inflammation, proliferation, and migration (50), and *Mapk14* deficiency in mice blocked cardiac fibroblast differentiation into myofibroblasts (51).

A key node gene found in our PPI network of the *Ace2* co-expressed genes is deubiquitinating enzyme cylindromatosis (*Cyld*), which is also identified as a candidate upstream regulator for *Ace2* by eQTL mapping. Although the regulatory relationship between *Cyld* and *Ace2* predicted in our study needs to be further validated, accumulating evidence suggests that *Cyld* is a crucial regulator of diverse cellular processes, such as immune responses, inflammation, death, and proliferation (52, 53). Cyld has a C-terminal catalytic domain characteristic of deubiquitinating enzymes, which is essential for Cyld to remove ubiquitin from certain proteins that positively mediate signaling through nuclear factor-kappa B (NF-kB) and JNK pathways (54); thus, Cyld acts a negative regulator of JNK and NF-kB signaling (55). *Cyld* is expressed in the intimal layer of the vessels as well as in cardiomyocytes. In the cardiomyocytes of hypertrophic and failing human and murine hearts, *Cyld* is highly upregulated, suggesting that *Cyld* may mediate cardiac injury and dysfunction during inflammation and vascular damage (55). Conversely, knockout of *Cyld* improved a survival rate and alleviated cardiac hypertrophy, fibrosis, apoptosis, oxidative stress, and contractile dysfunction in mice with sustained pressure overload after transverse aortic constriction (56). In this study, expression of *Cyld* was significantly negatively correlated with increased heart rate in BXD strains, further supporting the importance of *Cyld* in cardiac function.

Related to respiratory damages, our systems genetics approach found that levels of *Cyld*, but not *Ace2*, is correlated with increased circulation in pulmonary artery and related PH, evidenced by the fact that the BXD strains with lower *Cyld* expression have increased PV peak volume and PV peak pressure on echocardiograms. Echocardiographic signs of acute cor pulmonale, including RV dilation and dysfunction (RVD), paradoxical septal motion, and PH were the common symptoms in COVID-19 patients with acute respiratory distress (57). Moreover, PH, but not RVD, was associated with clinical signs of more severe COVID-19 and with worse in-hospital clinical outcome (58). Combining murine and human results, we suggest that CYLD may represent a novel target gene for PH and cardiopulmonary diseases. Interestingly, treatment with recombinant TNFα significantly increased CYLD expression in endothelial and vascular smooth muscle cells (59).

The protective effects of ACE2 and its product Ang1-7 against alveolar epithelial cell death by reducing JNK phosphorylation have been shown (60). The main cellular substrate activated by JNK-mediated phosphorylation is C-Jun encoded by *Jun* (61). Interestingly, our PPI network shows that C-Jun has the most connections with other genes. Ablation of *Jun* resulted in progressive myocardial fibrosis, cardiomyocyte apoptosis, and sarcomeric disorganization *in vivo*. These alterations were exacerbated in response to mechanical pressure overload resulting in premature heart failure (62), potentially explaining the genetic basis of cardiopulmonary complications in COVID-19 patients. Recently, supporting our murine results, the Human Coronavirus (HCoV)-host interactome network study has reported that JUN and NPM1 (both are found in our PPI network) are the important hub proteins selected out of 119 CoV-associated host proteins assembled using four known human (SARS-CoV, MERS-CoV, HCoV-229E, and HCoV-NL63), one mouse (MHV), and one avian (IBV) coronaviruses. These host proteins are either the direct targets of HCoV proteins or are involved in crucial pathways of HCoV infection (63). Although there are still many unknowns needed to validate in the future, we believe that our systems genetics approach based on *Ace2* and cardiovascular traits associations found in BXD mice provide important regulatory relationship between *Ace2* and the node genes, opening surplus avenues in hunting novel targets in cardiovascular and cardiopulmonary diseases, including cardiovascular complications in COVID-19 infection.

## Conclusion

In summary, our study aimed to understand the underlying genetic basis of cardiovascular complications through the dissection of Ace2 genetic correlates in murine GRP of BXD mice. We have found that *Ace2* expression is associated with systolic and diastolic BP, P-wave duration, and P-wave amplitude. *Ace2* co-varies with many genes that are enriched in pathways related to inflammation and cardiac damage, suggesting that all these novel genes (*Cyld, Jun, Mapk8*, and *Mapk14)* and pathways (RAS, TGFβ, TNFα, and p38α) can be targeted for preventive, diagnostic, and therapeutic purposes in patients with cardiac damage, including COVID-19 patients.

## Data Availability Statement

The gene expression data EPFL/LISP BXD CD Heart Affy Mouse Gene 2.0 ST (Jan14) RMA used in this study was generated through collaborative efforts and can be accessed at our GeneNetwork (GN) website (http://genenetwork.org/) with the GN485 accession number (http://gn1.genenetwork.org/webqtl/main.py?FormID=sharinginfo&GN_AccessionId=485) (22).

## Ethics Statement

The animal study was reviewed and approved by the Institutional Animal Care and Use Committee (IACUC) at the University of Tennessee Health Science Center (UTHSC).

## Author Contributions

LL and EP conceived the study. UM and NL conducted the experiments. FX and JG performed data analysis. FX, JG, LL, and EP wrote the manuscript, prepared the figures, and tables. UM, QG, JP, AS-D, JT, and YC edited the manuscript. All authors read and approved the final version of the manuscript for publication.

## Conflict of Interest

The authors declare that the research was conducted in the absence of any commercial or financial relationships that could be construed as a potential conflict of interest.
